# A Study of the Relationship Between Postoperative Complications of Acute Kidney Injury After Off-Pump Coronary Artery Bypass Grafting and the Control of Patients’ Blood Glucose Levels

**DOI:** 10.21470/1678-9741-2025-0367

**Published:** 2026-09-08

**Authors:** Xiaohu Han, Ting Xie, Yun Chen, Yan Zhou, Qinglu Wu

**Affiliations:** 1 Department of Cardiac Surgery, Hainan General Hospital (Hainan Affiliated Hospital of Hainan Medical University), Haikou, People’s Republic of China; 2 Department of Accounting, Hainan Women's and Children's Medical Center, Haikou, People’s Republic of China; 3 Department of Cardiovascular Medicine, Hainan General Hospital (Hainan Affiliated Hospital of Hainan Medical University), Haikou, People’s Republic of China

**Keywords:** Acute Kidney Injury, AKI, Coronary Artery Bypass, Off-Pump, Glucose Level, Extracorporeal Circulation.

## Abstract

**Objective:**

To explore the relationship between admission blood glucose and postoperative acute kidney injury (AKI) after off-pump coronary artery bypass grafting (OPCABG).

**Methods:**

One hundred seventy-seven patients who underwent OPCABG in the Cardiac Surgery Department of our hospital were selected for a retrospective study and were divided into 41 patients in the AKI group and 136 patients in the non-AKI group according to the occurrence of AKI in their postoperative period; the indexes of the patients at the time of admission were statistically analyzed using Student’s t-test for continuous variables and χ^2^ test for categorical variables; and the relationship between preoperative fasting plasma glucose (FPG) and the occurrence of AKI was explored by multivariable logistic regression analysis. Normality of continuous variables was assessed using the Shapiro-Wilk test.

**Results:**

The preoperative FPG and glycosylated hemoglobin (HbA1c) measurements of patients in the AKI group were higher than those in the non-AKI group, and the difference was statistically significant (P < 0.05); the results of the logistic regression model showed that elevated preoperative blood creatinine, elevated preoperative uric acid, elevated preoperative hyperglycemia, elevated preoperative HbA1c level, and elevated preoperative neutrophil-to-lymphocyte ratio value were the OPCABG surgery patients' independent risk factors for postoperative AKI (P < 0.05).

**Conclusion:**

Preoperative hyperglycemia - assessed by both FPG and HbA1c - is an independent risk factor for AKI after OPCABG. Clinicians should prioritize glycemic control before surgery to reduce AKI risk.

## INTRODUCTION

**Table:** 

Abbreviations, Acronyms & Symbols				
AKI	= Acute kidney injury		KDIGO	= Kidney Disease: Improving Global Outcomes
BMI	= Body mass index		LVEF	= Left ventricular ejection fraction
CABG	= Coronary artery bypass grafting		NLR	= Neutrophil-to-lymphocyte ratio
CI	= Confidence interval		OPCABG	= Off-pump coronary artery bypass grafting
CPB	= Cardiopulmonary bypass		OR	= Odds ratio
FPG	= Fasting plasma glucose		SE	= Standard error
HbA1c	= Glycosylated hemoglobin		VIF	= Variance inflation factor
ICU	= Intensive care unit			

Off-pump coronary artery bypass grafting (OPCABG) is a widely used surgical treatment for severe coronary artery disease. This procedure restores myocardial blood supply by bypassing occluded coronary arteries. However, it carries a risk of complications, particularly acute kidney injury (AKI). AKI can result from various factors, including intraoperative hemodynamic changes, contrast media use, infections, and drug reactions, leading to prolonged hospitalization, increased costs, and potential long-term health deterioration. Glycemic control is crucial in cardiac surgery, as hyperglycemia is a key risk factor for postoperative complications, including infections and impaired healing. Therefore, effective glucose management during OPCABG is essential to reduce the risk of complications such as AKI. Investigating the relationship between AKI after OPCABG and blood glucose levels is not only clinically relevant but may also reveal effective preventive strategies to reduce AKI incidence and improve patient outcomes. A deeper understanding of how blood glucose levels affect renal health and surgical recovery can guide more precise patient management. Recent studies (2020 - 2025) have further emphasized the role of hyperglycemia as a modifiable risk factor for AKI in cardiac surgery, supporting the need for stringent glycemic control protocols^[1,2]^.

Coronary atherosclerosis, which reduces myocardial blood supply, can lead to severe cardiac events, including sudden cardiac death. Surgical revascularization via coronary artery bypass grafting (CABG) is an established treatment for alleviating myocardial ischemia^[ 3,4 ]^. CABG remains a primary treatment for coronary artery disease^[5]^, and OPCABG is considered to offer significant advantages over on-pump CABG^[6]^. Cardiac surgery, however, is frequently complicated by AKI, which adversely affects patient prognosis. Therefore, identifying risk factors for AKI is essential for improving postoperative quality of life^[7]^. This study aims to investigate the relationship between admission blood glucose levels (assessed by both fasting plasma glucose [FPG] and glycosylated hemoglobin [HbA1c]) and the occurrence of postoperative AKI in a dedicated cohort of patients undergoing OPCABG, providing focused insights into glycemic risk in this specific surgical context.

## METHODS

### Patients

A post-hoc power analysis was performed using G*Power software (version 3.1.9.7) after complete data collection. Based on the observed data from the final 177-patient sample, which showed a mean FPG difference of 0.46 mmol/L between AKI and non-AKI groups with a standard deviation of 0.55, the analysis indicated that the study had 80% power to detect a statistically significant difference (α = 0.05, two-tailed). One hundred seventy-seven patients who underwent OPCABG surgery in the Cardiac Surgery Department of our hospital were selected for the retrospective study. All cases were planned as off-pump procedures. However, eight cases (4.5%) were converted to on-pump CABG due to hemodynamic instability. These converted cases were included in the primary analysis (intention-to-treat principle). A sensitivity analysis excluding these converted cases (n = 169) was performed, and the results are presented in the Results section (see Sensitivity Analysis), confirming the robustness of the primary findings. The patients were divided into 41 cases in the AKI group and 136 cases in the non-AKI group according to the occurrence of postoperative AKI. The time of patient collection was from January 2015 to January 2019. Inclusion criteria: (1) patients with elective surgery; (2) patients aged 19 - 79 years; (3) patients planned for OPCABG surgery; (4) the same group of healthcare professionals completed the OPCABG surgical operation; (5) the diagnostic criteria of AKI patients were referred to the diagnostic criteria proposed by the Kidney Disease: Improving Global Prognostic Organization (KDIGO) in 2013^[8]^; and (6) patients with complete essential data in the electronic medical record system. Exclusion criteria: (1) patients with renal dysfunction requiring hemodialysis before surgery; (2) patients with combined malignant tumors; (3) patients with emergency surgery; (4) patients with missing data, which made it impossible to complete the statistical analysis.

There were 41 cases in the AKI group, sex: 24 males and 17 females; age: 47 - 79 years old, mean 64.2 ± 8.3 years old; body mass index (BMI): 23.4 ± 2.2 kg/m^2^. There were 136 patients in the non-AKI group, sex: 86 males and 50 females; age: 45 - 79 years old, mean 65.4 ± 9.2 years old; patient BMI: 23.2 ± 2.5 kg/m^2^. Comparison of age, sex, and BMI between the two groups showed no statistically significant difference ( *P* > 0.05).

This study has been registered in the National Health Security Information Platform Medical Research Registration and Filing Information System with registration number: MR -46-23-022818, Registration platform website: https://www.medicalresearch.org.cn/login .

A detailed overview of the patient screening, exclusion, and inclusion process is provided inFigure 1.

**Fig. 1:**
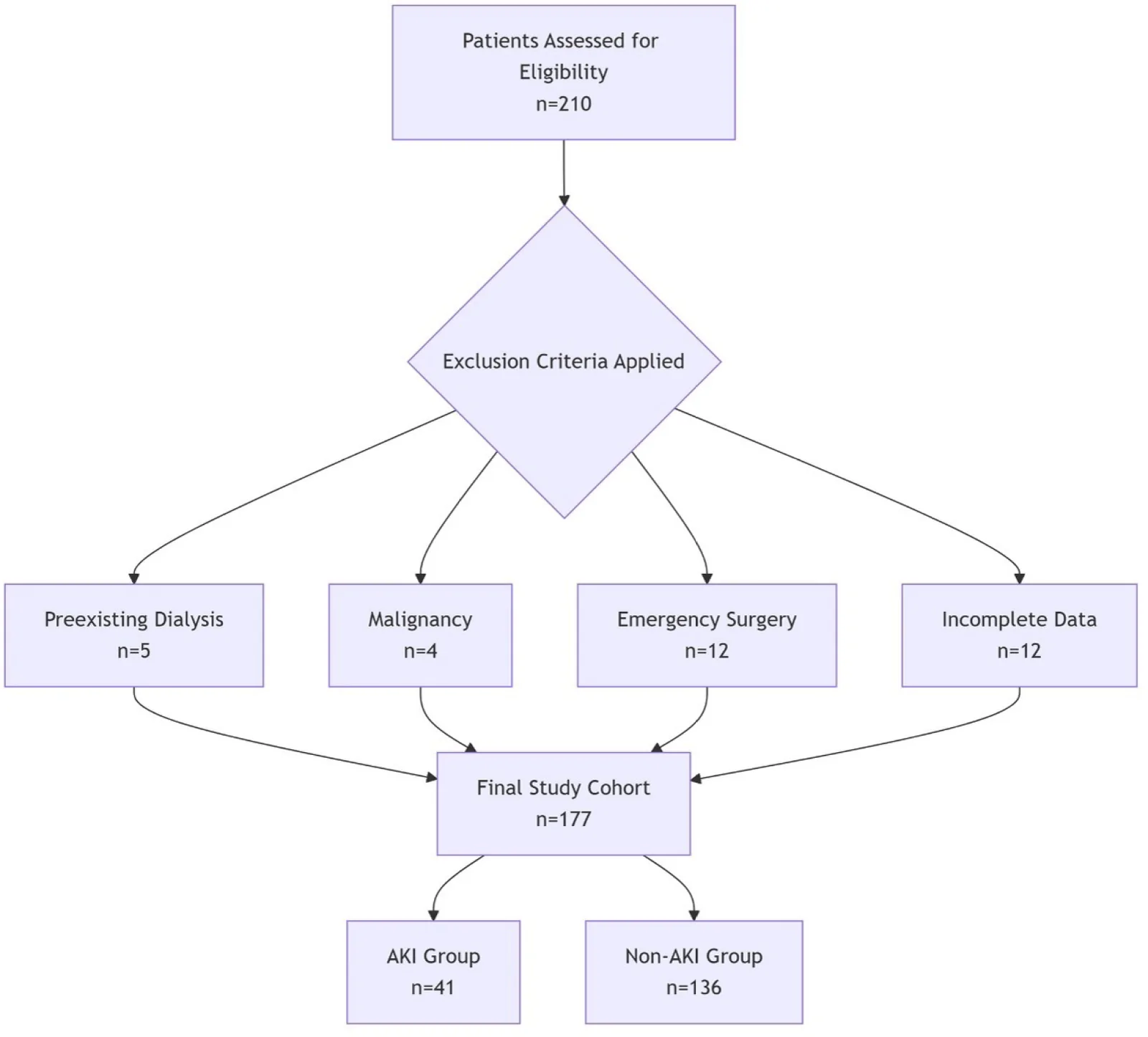
Participants’ flow diagram. This diagram illustrates the screening, exclusion, and final grouping process of patients undergoing off-pump coronary artery bypass grafting in this retrospective cohort study (January 2015 - January 2019). A total of 210 patients were initially assessed for eligibility. After exclusions (n = 33; preexisting dialysis: 5, malignancy: 4, emergency surgery: 12, incomplete data: 12), 177 patients were included in the final analysis and categorized into acute kidney injury (AKI) (n = 41) and non-AKI (n = 136) groups based on postoperative AKI occurrence.

### Surgical and Acute Kidney Injury Diagnostic Procedures

After general anesthesia and endotracheal intubation, the patient was placed in the supine position, a cut was made in the middle of the patient's sternum, the pericardium was opened to completely expose the patient's left anterior descending branch, the saphenous vein and the left internal thoracic artery were harvested, heparin was administered intravenously in a dosage of 1 mg/kg - 1.5 mg/kg, and then the patient was anastomosed to the target coronary arteries and the aorta. Then, the drainage tube was routinely placed, and the incision was closed one layer by one. Patients were subjected to patient-controlled intravenous analgesia with an infusion of fentanyl (10 µg/mg), a background dose of 20 µg/hour, and a patient-controlled dose of 2 ml for 15 minutes, which was discontinued 48 hours later. The patients were divided into AKI and non-AKI groups based solely on the KDIGO 2013 criteria^[8]^. AKI was defined and staged according to these criteria, using serum creatinine changes within 48 hours postoperatively. Urine output was not used for AKI diagnosis. Among the 41 AKI patients, the distribution of AKI stages was as follows: stage 1, n = 28 (68.3%); stage 2, n = 9 (22.0%); stage 3, n = 4 (9.8%).

### Methods of Clinical Indicators

Statistical analysis of FPG and HbA1c at admission was performed and compared. Age, sex, BMI, history of comorbidities, duration of surgery, intraoperative blood transfusion, number of diseased blood vessels, intensive care unit (ICU) stay, preoperative blood creatinine, preoperative uric acid, preoperative left ventricular ejection fraction (LVEF), and neutrophil-to-lymphocyte ratio (NLR) were collected.

All patients underwent blood glucose tests and HbA1c tests at the time of admission, which was defined as the morning of the day of surgery after an overnight fast. 3 mL of venous blood was collected from patients on fasting, 2 mL of ethylenediaminetetraacetic acid anticoagulated blood was collected, FPG was measured by glucose oxidase method, and HbA1c was measured by immunoturbidimetric assay. Blood creatinine and blood uric acid levels were detected by chemiluminescence and enzyme-linked immunosorbent assay, respectively. All patients underwent echocardiography to assess LVEF on admission, respectively.

### Institutional Protocols

During the study period, there was no standardized institutional protocol for perioperative glycemic management or renal protection. Glycemic control and renal protective measures were administered at the discretion of the attending anesthesiologist and surgeon.

### Statistical Analysis

Data analysis was performed using IBM SPSS Statistics for Windows, version 21.0 (IBM Corp., Armonk, N.Y., USA). The normality of continuous variables was assessed using the Shapiro-Wilk test. Normally distributed measurement data such as age, preoperative FPG, and HbA1c were expressed as mean ± standard deviation (x̅ ± s) and compared using Student's *t* -test for between-group analysis; non-normally distributed data were expressed as median (interquartile range) and compared using the Mann-Whitney U test. Categorical data such as sex and comorbidities were expressed as counts (percentages) and compared using χ^2^ test or Fisher's exact test as appropriate. Multicollinearity among independent variables in the logistic regression model was assessed using variance inflation factor (VIF). As this was a retrospective study, patients with missing data that precluded complete statistical analysis were excluded per the exclusion criteria (n = 0 for all key variables in the final analyzed cohort). Therefore, no missing data handling methods were required, and complete-case analysis was inherently employed. *P* < 0.05 was taken as the statistically significant difference. Cardiopulmonary bypass (CPB) time was not included in the multivariable logistic regression model due to its limited availability (only applicable to the eight converted patients), which would lead to unreliable estimates and model instability.

## RESULTS

### Comparison of Fasting Plasma Glucose and Glycosylated Hemoglobin Measurements in the Acute Kidney Injury and Non-Acute Kidney Injury Groups

The preoperative FPG (Figure 2) and HbA1c (Figure 3) measurements of patients in the AKI group were higher than those in the non-AKI group, and the difference was statistically significant ( *P* < 0.05,Table 1).

**Table 1:** Comparison of FPG and HbA1c measurements in AKI and non-AKI groups (x̅ ± s).

Groups	n	FPG (mmol/L)	HbA1c (%)
AKI group	41	5.64 ± 0.62	7.37 ± 1.66
Non-AKI group	136	5.18 ± 0.55	6.61 ± 1.43
*t*		4.555	2.871
*P* -value		< 0.001	0.005

AKI=acute kidney injury; FPG=fasting plasma glucose; HbA1c=glycosylated hemoglobin

**Fig. 2:**
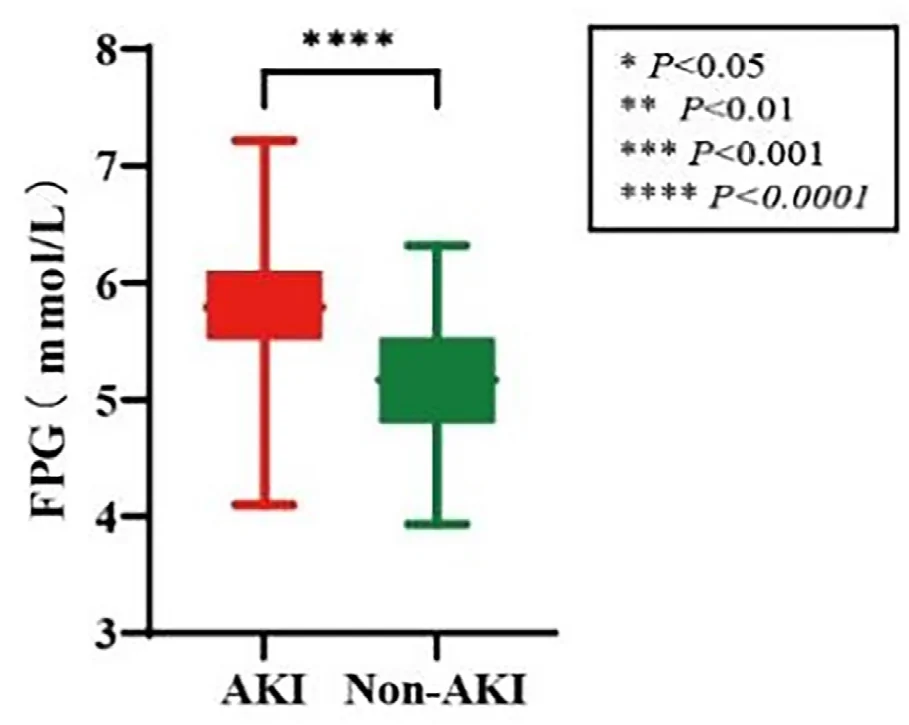
Comparison of fasting plasma glucose (FPG) levels in acute kidney injury (AKI) and non-AKI groups. Bar graph showing FPG levels (mean ± standard deviation) in the AKI (n = 41) and non-AKI (n = 136) groups. FPG was significantly higher in the AKI group (5.64 ± 0.62 mmol/L) compared to the non-AKI group (5.18 ± 0.55 mmol/L; P < 0.001, Student's t-test).

**Fig. 3:**
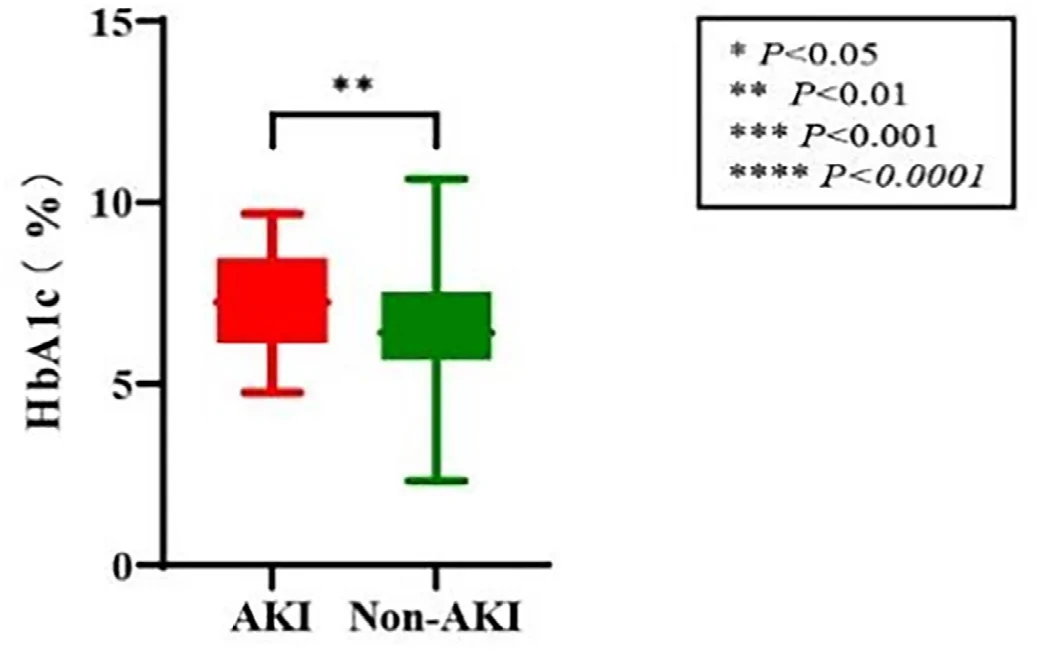
Comparison of glycosylated hemoglobin (HbA1c) levels in acute kidney injury (AKI) and non-AKI groups. Bar graph showing HbA1c levels (mean ± standard deviation) in the AKI (n = 41) and non-AKI (n = 136) groups. HbA1c was significantly higher in the AKI group (7.37 ± 1.66%) compared to the non-AKI group (6.61 ± 1.43%; P = 0.005, Student's t-test).

### Comparison of General Information Between Acute Kidney Injury and Non-Acute Kidney Injury Groups

Comparison of age, sex, BMI, combined hypertension, dyslipidemia, smoking, and alcohol consumption between patients in the AKI group and the non-AKI group showed no statistically significant differences ( *P* > 0.05,Table 2).

**Table 2:** Comparison of general information between AKI and non-AKI groups.

Feature	AKI group (n = 41)	Non-AKI group (n = 136)	*t* /χ^2^	*P* -value
Age (years)	64.2 ± 8.3	65.4 ± 9.2	-0.748	0.455
BMI (kg/m^2^)	23.4 ± 2.2	23.2 ± 2.5	0.461	0.645
Sex (%)			0.296	0.587
Male	24 (58.54)	86 (63.24)		
Female	17 (41.46)	50 (36.76)		
High blood pressure (%)			0.909	0.340
Yes	11 (26.83)	27 (19.85)		
No	30 (73.17)	109 (80.15)		
Dyslipidemia (%)			0.679	0.410
Yes	25 (60.98)	73 (53.68)		
No	16 (39.02)	63 (46.32)		
Cigarette smoking (%)			0.543	0.461
Yes	18 (43.9)	51 (37.5)		
No	23 (56.1)	85 (62.5)		
Drinking (%)			1.137	0.286
Yes	16 (39.02)	41 (30.15)		
No	25 (60.98)	95 (69.85)		

AKI=acute kidney injury; BMI=body mass index

### Comparison of Surgical Procedures and Laboratory Indexes Between Acute Kidney Injury and Non-Acute Kidney Injury Groups

Comparison of surgical time, preoperative hemoglobin level, number of branches of diseased vessels, ICU stay time, and preoperative LVEF measurements between patients in the AKI group and non-AKI group showed no statistically significant difference ( *P* > 0.05,Table 3); comparison of intraoperative transfusion rate CPB time (applicable only to the eight converted patients), preoperative blood creatinine, preoperative uric acid, preoperative albumin, preoperative NLR, and preoperative bilirubin level between patients in AKI and non-AKI groups showed that differences were statistically significant ( *P* < 0.05,Table 3). Among the 177 patients planned for OPCABG, eight (4.5%) required conversion to on-pump CABG due to hemodynamic instability.

**Table 3:** Comparison of surgical procedures and laboratory indices in the AKI and non-AKI groups.

Observation indicators	AKI group (n = 41)	Non-AKI group (n = 136)	t/χ^2^	*P* -value
Operating time (min)	297.8 ± 32.7	303.8 ± 30.0	-1.099	0.273
Intraoperative blood transfusion (%)			6.490	0.011
Yes	6 (14.63)	5 (3.68)		
No	35 (85.37)	131 (96.32)		
Preoperative hemoglobin (g/L)	123.6 ± 12.4	126.0 ± 11.6	-1.143	0.255
Preoperative albumin (g/L)	36.2 ± 3.8	39.3 ± 4.1	-4.314	< 0.001
Preoperative bilirubin (µmol/L)	29.71 ± 4.41	25.85 ± 4.78	4.612	< 0.001
Preoperative NLR	6.49 ± 1.80	4.20 ± 1.43	8.442	< 0.001
Number of diseased vessels (branches)	3.05 ± 0.85	2.98 ± 1.03	0.396	0.692
ICU stay (h)	43.8 ± 9.4	41.3 ± 10.0	1.422	0.157
Preoperative blood creatinine (µmol/L)	85.2 ± 16.3	76.1 ± 13.0	3.695	< 0.001
Preoperative uric acid (µmol/L)	366.8 ± 60.0	327.1 ± 55.4	3.945	< 0.001
Preoperative LVEF (%)	60.3 ± 5.9	61.0 ± 5.7	-0.684	0.495

AKI=acute kidney injury; ICU=intensive care unit; LVEF=left ventricular ejection fraction; NLR=neutrophil-to-lymphocyte ratio

### Multifactorial Analysis

A logistic regression model was established using preoperative FPG, HbA1c, intraoperative transfusion, preoperative blood creatinine, preoperative uric acid, preoperative albumin, preoperative NLR, and preoperative bilirubin measurements with statistically significant one-way analyses as independent variables, and whether or not AKI had occurred as a dependent variable. CPB time was not included in the model due to the small number of patients with available data (n = 8). Elevation of preoperative blood creatinine, preoperative uric acid, preoperative hyperglycemia elevation, elevated preoperative HbA1c level, and elevated preoperative NLR value were independent risk factors for postoperative AKI in patients undergoing OPCABG surgery ( *P* < 0.05,Table 4). Assessment of multicollinearity showed VIF values ranging from 1.12 to 2.45, with a maximum VIF of 2.45, confirming the absence of significant collinearity among the predictors.

**Table 4:** Logistic multifactor analysis results.

Parameters	bβ	SE	Walds	*P* -value	OR	95% CI	
Intraoperative blood transfusion	1.303	0.774	2.834	0.098	3.680	0.807	16.778
Preoperative blood creatinine	0.711	0.228	9.722	< 0.001	2.036	1.302	3.183
Preoperative uric acid	0.681	0.271	6.315	0.011	1.976	1.162	3.361
FPG	0.441	0.214	4.247	0.048	1.554	1.022	2.364
HbA1c	0.724	0.308	5.526	0.032	2.063	1.128	3.772
Preoperative albumin	-0.744	0.453	2.697	0.128	0.475	0.196	1.155
Preoperative bilirubin	0.398	0.273	2.125	0.195	1.489	0.872	2.542
Preoperative NLR	0.661	0.226	8.554	< 0.001	1.937	1.244	3.016

CI=confidence interval; FPG=fasting plasma glucose; HbA1c=glycosylated hemoglobin; NLR=neutrophil-to-lymphocyte ratio; OR=odds ratio; SE=standard error

### Clinical Outcomes

In-hospital mortality occurred in two patients (4.9%) in the AKI group and one patient (0.7%) in the non-AKI group ( *P* = 0.142, Fisher's exact test). Thirty-day mortality data were not collected. Renal replacement therapy was required in three patients (7.3%) in the AKI group and none in the non-AKI group ( *P* = 0.014, Fisher's exact test). ICU length of stay was 43.8 ± 9.4 hours in the AKI group and 41.3 ± 10.0 hours in the non-AKI group (mean difference: 2.5 hours; 95% confidence interval [CI]: -0.9 to 5.9 hours; *P* = 0.157, Student's *t* -test). Total hospital length of stay was 12.5 ± 3.2 days in the AKI group and 10.1 ± 2.8 days in the non-AKI group (mean difference: 2.4 days; 95% CI: 1.3 to 3.5 days; *P* < 0.001, Student's *t* -test). Other major postoperative complications ( *e.g.* , stroke, myocardial infarction) were not systematically collected.

### Diabetes Status and Exploratory Subgroup Analysis

This exploratory analysis was underpowered to detect statistically significant differences between diabetes subgroups. A prior diagnosis of diabetes mellitus was present in 18 patients (43.9%) in the AKI group, and 42 patients (30.9%) in the non-AKI group. Diabetes status was not included in the multivariable logistic regression model due to collinearity with FPG and HbA1c.

Among the 177 patients, 60 (33.9%) had a prior diagnosis of diabetes mellitus, and 117 (66.1%) did not. The incidence of AKI was higher in diabetic patients (30.0%, 18/60) compared to non-diabetic patients (19.7%, 23/117), but the difference was not statistically significant (χ^2^ = 2.45, *P* = 0.117). In diabetic patients, preoperative FPG and HbA1c levels were significantly higher in the AKI subgroup compared to the non-AKI subgroup (FPG: 5.82 ± 0.68 *vs.* 5.45 ± 0.59 mmol/L, *P* = 0.021; HbA1c: 7.85 ± 1.72 *vs.* 7.02 ± 1.58%, *P* = 0.045). In non-diabetic patients, preoperative FPG was also higher in the AKI subgroup (5.48 ± 0.55 *vs.* 5.10 ± 0.51 mmol/L, *P* = 0.003), but HbA1c did not differ significantly (6.42 ± 1.21 *vs.* 6.25 ± 1.35%, *P* = 0.589). These exploratory findings suggest that hyperglycemia may be a risk factor for AKI regardless of diabetes status, with a potentially more pronounced effect in diabetic patients.

### Sensitivity Analysis

A sensitivity analysis was performed excluding the eight patients who were converted from OPCABG to on-pump CABG (n = 169). The results of the multivariable logistic regression model for this cohort were consistent with the primary analysis. Elevated preoperative FPG (adjusted odds ratio [OR]: 1.52, 95% CI: 1.01 - 2.29, *P* = 0.046), HbA1c (adjusted OR: 2.01, 95% CI: 1.10 - 3.68, *P* = 0.024), blood creatinine (adjusted OR: 2.05, 95% CI: 1.31 - 3.21, *P* = 0.002), uric acid (adjusted OR: 1.95, 95% CI: 1.14 - 3.32, *P* = 0.014), and NLR (adjusted OR: 1.92, 95% CI: 1.23 - 2.99, *P* = 0.004) remained independent risk factors for postoperative AKI.

## DISCUSSION

The precise mechanism of AKI after CABG remains incompletely understood and varies across studies. Postoperative AKI adversely affects recovery quality following OPCABG, highlighting the need for enhanced preventive measures^[9 ]^.

Our findings reinforce that both acute and chronic hyperglycemia at admission are independent risk factors for AKI even in a planned OPCABG cohort. This underscores the critical role of glycemic control in this surgical setting, which is often perceived as lower risk for renal complications. The association of preoperative FPG with AKI risk in non-diabetic patients highlights a potential area for broader interventional strategies.

Some studies have found that elevated blood glucose is associated with renal function abnormalities after coronary angiography or cardiac surgery^[10]^. However, there are few reports on the relationship between blood glucose levels at admission and the occurrence of postoperative AKI in patients with OPCABG. The level of FPG reflects the body's instantaneous blood glucose level, which is affected by physiological and pathological factors^[11]^. HbA1c reflects the long-term level of glycemic control, and the body's blood glucose level increases HbA1c measurement which is not easily affected by external factors and can compensate for the instability of blood glucose measurement alone^[12]^. When patients are chronically hyperglycemic, the kidneys exhibit varying degrees of microvascular damage due to chemical and vascular structural changes that result in intercellular proliferation, glomerular basement membrane thickening, vascular endothelial damage, and increased permeability^[13]^. The results of this study showed that preoperative FPG and HbA1c values were significantly higher in the AKI group than in the non-AKI group and that preoperative hyperglycemia and elevated HbA1c levels were independent risk factors for postoperative AKI in patients undergoing OPCABG surgery. Studies have shown that the occurrence of AKI is also related to factors such as oxidative stress, inflammatory response, and endothelial function of the body^[14]^. When analyzed in conjunction with the results of this study, preoperative hyperglycemia increases the degree of these responses, induces apoptosis and platelet aggregation, and ultimately provides conditions for the development of AKI. Furthermore, hyperglycemia may promote the formation of advanced glycation end-products, which can impair renal autoregulation by altering vascular reactivity and promoting inflammatory pathways. These mechanisms collectively contribute to renal injury in the context of cardiac surgery. Consistent with our findings, recent guidelines and cohort studies (2020 - 2025) highlight that hyperglycemia and poor glycemic control are independent predictors of AKI after cardiac surgery and recommend preoperative HbA1c screening and perioperative glucose management to mitigate renal injury^[15,16]^. Practical implications include preoperative glycemic optimization through lifestyle interventions or medication adjustments and targeting intraoperative blood glucose levels below 180 mg/dL (10 mmol/L) to reduce AKI risk, although causality cannot be confirmed due to the observational nature of this study.

The OPCABG procedure does not require cross-clamping of the ascending aorta. OPCABG is closer to the physiologic state than cardiac perfusion, which improves systemic inflammatory response without perfusion through cardioplegic arrest. The results of this study suggest that differences in age, sex, BMI, comorbid hypertension, dyslipidemia, operative time, number of branches of diseased vessels, ICU stay, and preoperative LVEF measurements are not risk factors for the development of AKI, probably because of the small sample size of this study, which biased the results, and the effect of these factors on the prognosis of the patients was not observed.

Serum creatinine, filtered by glomeruli and excreted in the urine, is an essential indicator for assessing renal function^[17]^. High preoperative levels of blood creatinine can increase the likelihood of renal injury in patients after surgery. Serum creatinine levels increase significantly when the glomerular filtration rate is reduced to about half the average level. Therefore, if preoperative serum creatinine levels are higher than usual, it indicates impaired renal function^[18]^. When the heart is exposed and the proximal vessels are manipulated during OPCABG, the decrease in the body's blood pressure and the increase in the venous pressure can cause the kidneys to show hypoperfusion^[19]^. When vasoactive drugs are used, for example, the body releases a large amount of inflammatory factors and oxygen radicals, which can cause damage to the proximal small vessels of the kidneys. It has been reported that serum creatinine has value in the early diagnosis of AKI, and the mechanism of AKI after OPCABG may be related to intraoperative moving of the heart, hemodynamic fluctuations, and stress reactions that affect renal perfusion^[20]^.

Serum uric acid is significantly elevated in the serum of patients with renal dysfunction; therefore, it can be used as an indicator for preoperative assessment of renal function^[21,22]^, and the exact reason for the development of AKI after surgery is not fully understood. The results of this study showed that increased preoperative uric acid levels can increase the risk of postoperative AKI. CPB time can likewise be used as a risk factor for AKI after OPCABG. The reason for this analysis is that, as the surgery proceeds, the surgical stimulus becomes progressively more injurious to the patient, which in turn activates the body's inflammatory response, resulting in the release of a large number of inflammatory factors, which leads to an increase in the incidence of AKI.

Our subgroup analysis by diabetes status revealed that the incidence of AKI was higher in diabetic patients, though not statistically significant, possibly due to the limited sample size. Importantly, hyperglycemia, as measured by FPG and HbA1c, was associated with AKI risk in both diabetic and non-diabetic subgroups, reinforcing the importance of glycemic control in all patients undergoing OPCABG.

### Limitations

This study has several limitations that should be considered. First, its retrospective and single-center design is inherently susceptible to selection and information bias, despite our efforts to include consecutive patients and complete data. The findings may not be fully generalizable to other populations or institutions with different patient demographics or perioperative protocols. Second, the sample size calculation was performed as a post-hoc analysis based on the final dataset; although the observed power was 80%, the relatively small number of AKI events (n = 41) may still limit the statistical power for some analyses and increases the risk of overfitting the multivariable model. Furthermore, data on long-term renal outcomes and major postoperative complications other than those reported were not systematically collected, restricting our analysis to short-term in-hospital outcomes. Finally, unmeasured confounding factors could have influenced the results.

## CONCLUSION

This study identified elevated preoperative FPG, HbA1c, blood creatinine, uric acid, and NLR as independent risk factors for AKI following OPCABG. These findings suggest that admission glycemic levels, both acute and chronic, are significantly associated with postoperative AKI risk. The results highlight the potential importance of preoperative assessment and optimization of these parameters, particularly glycemic control, in patients scheduled for OPCABG.

## Data Availability

The authors declare that the data will be available upon request to the authors.

## Artificial Intelligence Usage

The authors declare that no artificial intelligence tool was used in the preparation of this article.
